# Individual, social and physical environmental correlates of sedentary behaviours in adults: a systematic review protocol

**DOI:** 10.1186/2046-4053-3-120

**Published:** 2014-10-21

**Authors:** Stephanie A Prince, Katelin M Gresty, Jennifer L Reed, Erica Wright, Mark S Tremblay, Robert D Reid

**Affiliations:** 1 Division of Prevention and Rehabilitation, University of Ottawa Heart Institute, 40 Ruskin Street, Ottawa, Ontario K1Y 4W7, Canada; 2 School of Rehabilitation Sciences, University of Ottawa, 75 Laurier Ave East, Ottawa, Ontario K1N 6N5, Canada; 3 Berkman Library, University of Ottawa Heart Institute, 40 Ruskin Street, Ottawa, Ontario K1Y 4W7, Canada; 4 Healthy Active Living and Obesity Research Group, Children’s Hospital of Eastern Ontario Research Institute, 401 Smyth Road, Ottawa, Ontario K1H 8L1, Canada

**Keywords:** Sedentary behaviour, Socio-ecological correlates, Adults

## Abstract

**Background:**

Adults spend the majority of their time being sedentary, and evidence suggests that those who spend more of their day engaged in sedentary activities (TV viewing, sitting, screen-based activities) are at increased risk for morbidity and mortality, regardless of whether they exercise regularly. In order to develop effective interventions to reduce sedentary time, it is necessary to identify and understand the strongest modifiable factors of these behaviours. Therefore, the objective of this systematic review is to examine the available evidence in order to identify individual, social, environmental and policy correlates and determinants of sedentary behaviours (TV time, sitting time, screen time) and total sedentary time among adults.

**Methods/design:**

Six electronic databases will be searched to identify all studies that report on individual, social and/or environmental correlates and determinants of sedentary behaviours and total sedentary time in adults. Grey literature sources including theses, published conference abstracts and websites from relevant organizations will also be included. Articles that report on modifiable individual (e.g. health behaviours and status, self-efficacy, socio-economic status), social (e.g. crime, safety, social support, climate and capital), environmental (e.g. weather, workplace, home, neighbourhood, recreation environment, transportation environment) and policy correlates and determinants (based on study design) of sedentary behaviours in an adult population (mean age ≥18 years) will be included. Study quality and risk of bias will be assessed within and across all included studies. Harvest plots will be used to synthesize results across all correlates, and meta-analyses will be conducted where possible among studies with sufficient homogeneity.

**Discussion:**

This review will provide a comprehensive examination of evidence in the field and will serve to highlight gaps for future research on the determinants of sedentary behaviours and inform intervention design.

**Systematic review registration:**

PROSPERO CRD42014009814

## Background

Epidemiological studies have shown that adults spend the majority of their day being sedentary [1,2]. Sedentary behaviours are defined as a low energy expenditure while in a sitting or reclining posture during waking hours and are distinct from the simple absence of physical activity [3]. Research consistently identifies various sedentary behaviours (e.g. total sitting time, TV viewing time, screen time) as well as device-based total sedentary time (e.g. accelerometer-derived) as independent risk factors for weight gain, chronic diseases including heart disease, diabetes and cancers, as well as premature mortality [4,5]. Further, a gradient effect exists with greater risks for morbidity and mortality among those who spend more of their day being sedentary, regardless of whether they engage in regular moderate-to-vigorous intensity physical activity (MVPA) [4,6-10].

**Figure 1:**
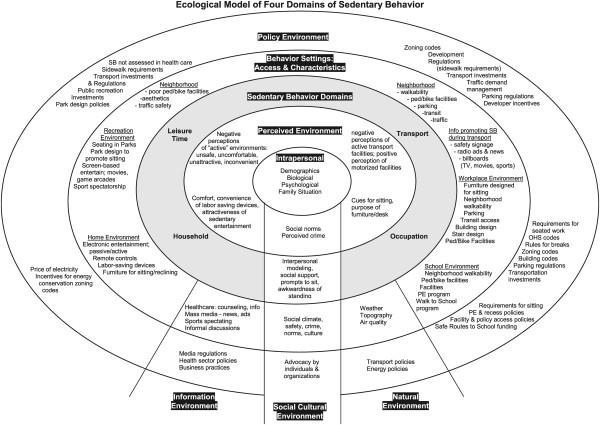
**Ecologic model of four domains of sedentary behaviour **[16]** (reproduced with permission from the author and the publisher).**

A variety of factors are likely to influence an individual’s choice to engage in sedentary behaviours. Socio-ecology theory recognizes that individual behaviours may be dependent on the dynamic relationships between multiple determinants (e.g. biology, motivation, self-efficacy, socio-cultural, policy, built and natural environments) across several levels (e.g. intrapersonal, interpersonal, community) [11]. The theory has been widely applied to research looking at determinants of physical activity behaviours [12-15]. Building upon its past use in physical activity research, Owen et al. proposed an ecological model of sedentary behaviours as a conceptual approach to understanding the determinants of time spent in these behaviours across different domains (i.e. leisure time, transport, household, and occupation) (Figure 1) [16]. Their model provides a schematic framework that recognizes the possible behaviour settings and contextual factors that have the capacity to influence time spent in these sedentary behaviours.

To develop effective interventions and appropriate policies, it is necessary to identify and understand possible modifiable factors that can influence an individual’s sedentary behaviour habits. In their review, Owen and colleagues also examined the status of the literature and suggested five research priorities to advance knowledge around determinants and interventions of sedentary behaviours. Included in these priorities was a call for further research to establish correlates especially at multiple levels of influence [16]. Recently, Rhodes et al. conducted a systematic review that largely focused on individual and intrapersonal correlates of sedentary behaviours [17]. Their review identified several socio-demographic and health factors related to sedentary behaviours that were largely non-modifiable and did not examine correlates beyond the level of the individual such as factors in the social, built and physical environments, policies or several other important intrapersonal factors (e.g. self-efficacy, motivation, health status). Since the publication of these reviews, there have been a large number of studies that have reported on various correlates of sedentary behaviours across multiple levels of influence.

In addition to the growing body of evidence around correlates of sedentary behaviours and time spent sedentary, interventions have begun to emerge looking at strategies to reduce sedentary time among adults [18]. Although interventions are being proposed and tested, there has been relatively little work examining and synthesizing the available evidence to identify the strongest correlates and determinants of domain-specific sedentary behaviours and/or total sedentary time which are likely to be the best targets for interventions. It is therefore imperative that the most important factors affecting sedentary behaviours are identified for the development of effective interventions and appropriate policies. There is currently a need for a comprehensive synthesis of the available evidence to inform future research and intervention development. In response to this need, the objective of the proposed systematic review is to identify individual, social and environmental correlates and determinants of domain-specific sedentary behaviours (e.g. TV viewing time, leisure-based screen time, office-based screen time, office-based sitting time and time spent travelling in cars, buses, etc.) and total sedentary time among adults.

## Methods/design

### Study design

A systematic review and meta-analysis will be undertaken to identify significant individual, social and environmental correlates and determinants of domain-specific sedentary behaviours and total sedentary time in adults. The systematic review will adhere to the reporting guidelines of the *Preferred Reporting Items for Systematic Reviews and Meta-Analyses* (PRISMA) statement [19] and will meet the items outlined in *A Measurement Tool to Assess Systematic Reviews* (AMSTAR) checklist [20,21].

### Study registration

This systematic review is prospectively registered with PROSPERO (registration number CRD42014009814; http://www.crd.york.ac.uk/PROSPERO).

### Criteria for considering studies for this review

#### Type of studies

Studies will be included irrespective of their publication status. In order to increase external validity of the findings and due to the difficulty in modifying an individual’s exposure to intrapersonal and environmental factors, the systematic review will include all observational (prospective cohort, cross-sectional and case studies) and experimental (randomized controlled trials (RCTs), pre-post design, quasi-experimental) studies that quantify the association between a risk factor/correlate/determinant and domain-specific sedentary behaviours and total sedentary time in adults. Depending on the number of studies identified, a further exclusion criterion related to study design (only prospective or experimental designs) may be applied as these designs would enable the identification of determinants rather than associations. If there is an adequate number of RCTs, a summary of this evidence and the confidence in this evidence using Cochrane’s Grades of Recommendation, Assessment, Development and Evaluation (GRADE) approach [22] will be provided to increase internal validity of the review.

#### Types of participants

Studies will be included if the population is identified as being comprised mainly of adults with a mean age of 18 years or older. Population characteristics will be extracted for possible subgroup analyses (e.g. younger versus older, males versus females, special populations such as occupations and disease status). Studies with a mean age lower than 18 years or in non-humans (e.g. rats) will be excluded. Depending on the number of studies identified for certain subpopulations, the review may be divided into multiple papers to capture correlates and determinants relevant to specific populations (e.g. cardiac patients, elderly).

#### Types of exposures

Using an ecological model of health behaviours [11], the review will examine all modifiable intrapersonal (e.g. health status, self-efficacy, working status, socio-economic status, family status), social environmental (e.g. crime, safety, social support, social climate, social capital), physical environmental (e.g. weather, workplace environment, home environment, neighbourhood design, recreation environment, transportation environment) and policy environment correlates and determinants (depending on study design) of domain-specific sedentary behaviours (e.g. TV viewing, leisure-based screen time, office-based screen time, office-based sitting time and time spent travelling in cars, buses, etc.), as well as total daily time spent being sedentary (minutes/day). The ecological model of sedentary behaviour recognizes that domain-specific sedentary behaviours are largely influenced from factors in the behaviour settings themselves, including the neighbourhood, recreation environment, home environment, workplace environment, school environment and transportation environment. Further, the social-cultural environment, policy environment, information environment and natural environments are recognized as playing a role within each of these domains and settings. The exposures will be identified as either objectively measured (e.g. crime rate in a neighbourhood) or perceived factors (e.g. feelings of safety in a neighbourhood).

#### Types of comparators

Comparator or control groups are largely foreseen as not applicable in this research as most research designs are likely to be either cross-sectional, case-controls or prospective cohorts. However, in the case of possible randomized controlled studies (including cluster RCTs), control or usual care groups will consist of individuals not exposed to one of the individual, social or environmental factors.

#### Types of outcome measures

Measures of association and risk between an exposure and a sedentary behaviour or sedentary time will be captured from all studies. These outcomes will include correlation statistics, odds ratios and relative risks. Further, measures of time spent in sedentary behaviours (e.g. minutes per day spent sedentary, minutes per day watching TV) and, where possible, a measure of variance around the outcome (e.g. standard error, 95% confidence intervals) will be extracted from all eligible and included studies regardless of the unit of measurement or method of measurement. Sedentary behaviour is defined as a behaviour with an energy expenditure ≤1.5 metabolic equivalents of task (METs), where $1\;\mathrm{MET}\;=\;1\left(\frac{\mathrm{kcal}}{\mathrm{kg}*h}\right)$, while in a sitting or reclining posture during waking hours and not simply the absence of physical activity [3]. Sedentary behaviours can be either objectively measured (e.g., accelerometers, inclinometers, activity monitors, observed patterns) or self-reported (e.g., International Physical Activity Questionnaire, Sedentary Behaviour Index, Sedentary Behaviour Questionnaire). Further, sedentary behaviours can be described using a composite measure of total sedentary time or be domain specific (e.g. time spent watching TV, time spent sitting at work or home, time spent riding in a car and screen time (including computers and video games)). Depending on the number of studies identified for certain domains of sedentary behaviours, the review may be divided into multiple papers to capture correlates and determinants relevant to specific domains (e.g. TV viewing versus office sitting). Potential and known health sequelae of sedentary behaviours (e.g. obesity, diabetes) will not be extracted in this systematic review.

### Search methods for the identification of studies

A comprehensive search strategy has been designed by a research librarian (EW) and includes a search of six electronic databases: Ovid MEDLINE(R) In-Process (1946 to present), EBM Reviews—Cochrane Central Register of Controlled Trials (present), EMBASE Classic+ (1947 to present), Ovid PsycINFO (1806 to present), SPORTDiscus (1830 to present) and Dissertations and Theses (1861 to present). The strategy is illustrated using the Medline search as an example (Table 1) and was modified according to the indexing systems of the other databases. Grey literature (non-peer-reviewed works) that meets the inclusion criteria will be obtained including published conference abstracts indexed under the bibliographic databases, published lists of theses and dissertations, government reports and unpublished data and manuscripts (provided by original authors). Government reports will be searched using the Google search engine using a combination of key text words including “correlates or determinants of sedentary behaviours”. At the full-text screening phase, unpublished data and manuscripts will be solicited from authors of studies that report on collecting sedentary behaviours or sedentary time, but in which this data is not available within a published manuscript. For instance, if an abstract or paper reports on collecting physical activity data via accelerometer, we will inquire if sedentary time was also examined. Knowledgeable researchers in the field, including those affiliated with the Sedentary Behaviour Research Network (*http://www.sedentarybehaviour.org*), will be solicited for studies of interest. The bibliographies of studies selected for the review will also be examined to identify further studies. The Google search engine will be used to identify studies that are published in non-indexed journals.

**Table 1:** Medline search strategy

	Outcome terms
1	Sedentary lifestyle/
2	sedentary.tw.
3	((sitting or reclining) adj2 time).tw.
4	Physical* inactive*.tw.
5	screen time.tw.
6	((watch* or view*) adj (television or TV)).tw.
7	((television or TV) adj (viewing or watching or time)).tw.
8	(computer adj (time or "use")).tw.
9	(play* adj (videogame* or "video game*" or "computer game*" or "electronic game*")).tw.
10	((driving or commut*) adj2 time).tw.
11	Or/1-10
	Physical environment terms
12	Environment Design/
13	Residence Characteristics/
14	poverty areas/
15	built environment*.tw.
16	(walkable or walkability).tw.
17	(active adj (travel* or transportation or commut*)).tw.
18	((walking or pedestrian or cycling or bicycle or bike) adj (trail* or path* or route* or lane* or infrastructure)).tw.
19	((road or street) adj connectivity).tw.
20	(community adj2 (feature* or characteristic*)).tw.
21	community design.tw.
22	neighbo?rhood*.tw.
23	sidewalk*.tw.
24	green space.tw.
25	parks.tw.
26	Public Facilities/
27	Fitness Centers/
28	((sport* or recreation* or exercise) adj facilit*).tw.
29	("land use" adj2 mix*).tw.
30	(environment* adj (factor* or correlate* or determinant*)).tw.
31	Weather/
32	weather.tw.
33	(gym or gyms).tw.
34	((fitness or recreation*) adj (centre* or center*)).tw.
35	Or/12-34
	Social environment terms
36	Social Environment/
37	community networks/
38	Crime/
39	((safe* or unsafe) adj2 neighbo?rhood*).tw.
40	social support/
41	exp Socioeconomic Factors/
42	Culture/
43	Cultural Characteristics/
44	(social* adj (capital or support* or influence* or environment* or connect* or correlate* or factor*)).tw.
45	(socioeconomic or socio-economic).tw.
46	(sociodemographic* or socio-demographic*).tw.
47	(cultural adj (factor* or correlate* or influence*)).tw.
48	Or/36-47
	Policy terms
49	Public Policy/
50	health policy/
51	Or/49-51
	Intrapersonal terms
52	self efficacy/
53	motivation/
54	health status/
55	attitude to health/
56	health knowledge, attitudes, practice/
57	health behavior/
58	self efficacy.tw.
59	Motivation.tw.
60	Or/53-60
61	35 or 48 or 51 or 60
62	11 and 61
63	child/ not exp adult/
64	62 not 63
65	Adolescent/ not exp adult/
66	64 not 65

### Selection of studies

Articles will be imported into EndNote (Thompson Reuters, San Francisco, CA, USA) and duplicates removed using the “duplicate” function. Remaining duplicates will be removed manually. Two independent reviewers (SAP, KMG) will screen the titles and abstracts of all studies to identify potentially relevant articles. The full texts of all studies that either meet the inclusion criteria or provide insufficient information in the abstract to exclude will be obtained and reviewed. Two independent reviewers will screen the full texts for inclusion (SAP, KMG). If disagreements between the reviewers occur, consensus will be achieved through discussion with a third reviewer (JLR, MST or RDR). Reviewers will not be blinded to the authors or journals when screening articles.

### Data collection

Prior to data extraction, a data extraction form will be created and pilot-tested by the extractors using a subset of the included studies. The extraction form will be modified based on feedback from the extractors in order to improve its usability and ensure that complete and appropriate information is obtained. Standardized data abstraction forms including quality assessments will be completed by one reviewer (SAP) and verified by another (KMG). If disagreements occur, consensus will be achieved through discussion and/or with a third reviewer (JLR, MST or RDR). Reviewers will not be blinded to the authors or journals when extracting data.

From each included non-intervention study (e.g. prospective cohort, case-control, cross-sectional designs), the following information will be extracted: lead author; year of publication; country of study; participant characteristics (age range, sex distribution, health status, setting); sample size and study design; length of follow-up (if applicable); exposure/determinant/correlate (separate entry for each correlate examined); measurement method for each exposure/determinant/correlate (including whether it is self-reported or objectively measured); level of the correlate, e.g. individual, social environment, physical environment; whether sedentary behaviours are self-reported or objectively measured; sedentary behaviour measurement method and units of measurement; analytical methods used (e.g. bivariate, adjusted/multivariate); relationships between the exposure/determinant/correlate on sedentary behaviour (significant positive, negative or absence of association); and effect on sedentary behaviours (e.g. change in sedentary behaviours). Both unadjusted and adjusted results will be abstracted from the original studies, and meta-analyses will be conducted using the unadjusted results. Similar data will be extracted from intervention studies (RCT, quasi-RCT, pre-post design) but will also include intervention details, a description of the control group, information about blinding and randomization techniques, analytical methods used (e.g. *t*-test, adjusted/multivariate) and the effect of the intervention on sedentary behaviours (e.g. mean difference achieved, relative risks) including a measure of variance (95% confidence intervals or standard error/deviation). Authors of suspected duplications (report on same relationships between correlate/determinant and sedentary outcome) will be contacted, and in cases where several publications report the same results from the same data source, only one study per data source/analysis will be retained in order to avoid double counting.

If a paper employs a measure that has the potential to capture sedentary behaviours (e.g. International Physical Activity Questionnaire, accelerometers), but does not report on these outcomes in the manuscript, or if a paper reports on a study protocol, the authors will be contacted to ascertain whether the sedentary behaviour-related results can be provided. A maximum of three e-mail attempts will be made to contact the lead author of these studies to obtain additional information.

### Risk of bias and quality within studies

The quality of individual studies will be assessed using the Downs and Black checklist [23] and risk of bias assessed using the Cochrane Collaboration’s tool for assessing risk of bias in randomized trials [24]. The Downs and Black instrument to be employed in this review will assess study quality regardless of study design including reporting, external validity and internal validity (bias). The checklist consists of 27 items with a maximum count of 32 points. A modified version of the checklist will be employed with items that are not relevant to non-experimental studies review removed. The adapted checklist will consist of 19 items, including items 1–7, 9–13, 16–18, 20, 22, 26 and 27 from the original list, including 1 point for item #27 if the study was powered to determine the association between an exposure and sedentary behaviours. The maximum possible score for the modified checklist will be 19 points (higher scores indicate superior quality). The quality of individual studies will be rated by SAP and verified by KMG. The risk of bias assessment will be carried out for all experimental studies. Items included in Cochrane’s risk of bias assessment include sequence generation (randomization); allocation concealment; blinding of participants, personnel and investigator; incomplete data (e.g. losses to follow-up, intention-to-treat analysis); selective outcome reporting; and other possible sources of bias. The risk of bias assessment will be carried out by two independent assessors (SAP and KMG); if disagreements between assessors occur, consensus will be achieved through discussion with a third reviewer (JLR).

### Quality of the evidence

The quality of the evidence within each exposure (at individual, social and physical environment levels) will be assessed as high, moderate, low or very low using Cochrane’s GRADE approach [22]. Within this approach, RCTs begin as high-quality evidence and non-randomized studies begin as low-quality evidence. In addition to the study design, the quality of evidence will be rated upon possible risk of bias, imprecision, heterogeneity, indirectness or suspicion of publication bias. Risk of bias will be assessed using RevMan 5.2 and imported into GRADEpro Version 3.6 (GRADE Working Group) in order to rate the quality of the evidence using GRADE methodology.

### Analysis

Qualitative tables will be created to describe the populations, interventions (if applicable) and outcomes of all studies. This systematic review plans to assess relationships between a large number of exposures (at different levels, i.e. individual, neighbourhood) and sedentary behaviours which are likely assessed using a variety of methods. Due to the variety in the exposures and metrics used in the studies, the review will use harvest plots [25,26] as a method of synthesis. The harvest plots allow results of the primary studies to be displayed across the various exposures and metrics (e.g. perceived or objectively measured neighbourhood walkability) across various levels (i.e. individual, social environment, built environment, policy environment) and across various outcome measurement methods (i.e. self-reported vs. objectively measured sedentary behaviours) to incorporate the strength of association, sample size and study quality. The harvest plots will provide a graphical method to allow for a complete synthesis of the evidence and allow a comparison of the evidence across the various exposures.

Forest plots and meta-analyses will be created using Review Manager (RevMan) 5.2 (The Nordic Cochrane Centre, The Cochrane Collaboration, 2012) to synthesize the measures of effect (e.g. odds ratio, relative risk) and 95% confidence intervals for each exposure on sedentary behaviours. A random-effects meta-analysis will be used as effect sizes are likely to be similar, but not identical across all studies. Heterogeneity will be assessed using the *I*^2^ statistic with values above 75% and *p* <0.10 used to indicate high heterogeneity across studies [27]. Publication bias will be assessed using a funnel plot of the included studies’ estimates of effect. The plots will be assessed both visually and by using Egger’s test, with *p* <0.10 used to indicate the presence of a significant publication bias [28].

### Subgroup analyses

In addition to the primary analyses proposed, several *a priori* determined subgroup analyses will be performed when sufficient data are available. The analyses will examine differences between males and females, age groups (e.g. young, middle-aged and elderly adults), populations (e.g. healthy populations versus chronic disease populations), self-reported and objectively measured exposures, self-reported and objectively measured sedentary behaviours, studies with high and low risk of bias, different lengths of follow-up, trends over time (e.g. 1970s, 1980s, 1990s, 2000s) and unpublished versus published results.

## Discussion

This systematic review will be the first to critically examine and synthesize the available literature looking at the relationships between individual, social and environmental factors and adult sedentary behaviour. The review will provide a comprehensive examination of the evidence in the field to date and will serve to highlight gaps where future research on the determinants of sedentary behaviours will need to be conducted. The use of the harvest plots will allow researchers to visually examine all of the correlates across the multiple levels of influence that are related to sedentary behaviours including the strength and quality of the evidence. It is anticipated that this review will be useful for a variety of stakeholders including those looking to design interventions targeting the most important modifiable factors in order to reduce sedentary time.

## Abbreviations

AMSTAR: A Measurement Tool to Assess Systematic Reviews; GRADE: Grades of Recommendation, Assessment, Development and Evaluation; METs: metabolic equivalents of task; MVPA: moderate-to-vigorous intensity physical activity; PRISMA: Preferred Reporting Items for Systematic Reviews and Meta-Analyses; RCT: randomized controlled trial.

## Competing interests

The authors declare that they have no competing interests.

## Authors’ contributions

SAP conceived the study, design and methodology, provided input for the bibliographic search strategy, and drafted and edited the manuscript. JLR participated in its design and coordination and provided critical revision of the manuscript. KMG participated in its design and coordination and provided critical revision of the manuscript. EW conceived the bibliographic search strategy, participated in its design and provided critical revision of the manuscript. MST participated in the design of the study, provided expert input and critically reviewed the manuscript. RDR participated in the design of the study, provided methodological input and critically reviewed the manuscript. All authors read and approved the final manuscript.
